# Ultrasound-Assisted Extraction of Polysaccharides from *Lyophyllum decastes*: Structural Analysis and Bioactivity Assessment

**DOI:** 10.3390/molecules30040961

**Published:** 2025-02-19

**Authors:** Qiong Wu, Bin Liang, Jiaming Wang, Yonggang Dai

**Affiliations:** 1 College of Food Science and Engineering, Changchun University, Changchun 130012, China; 18088612573@163.com (B.L.); a1361002583@163.com (J.W.); 2 Jilin Academy of Agricultural Sciences, Changchun 130012, China; daiyonggang1976@163.com

**Keywords:** *Lyophyllum decastes*, polysaccharides, biological activity

## Abstract

This study employed ultrasound-assisted extraction (UAE) to isolate polysaccharides from *Lyophyllum decastes*, which were subsequently fractionated into two components, LDP-A1 and LDP-B1, using DEAE cellulose-52 and Sephacryl S-500. The structural characteristics of the polysaccharides were preliminarily analyzed using high-performance liquid chromatography (HPLC), Fourier-transform infrared (FTIR) spectroscopy, scanning electron microscopy (SEM), X-ray diffraction (XRD), and Congo red staining. The results indicate significant differences between LDP-A1 and LDP-B1 in terms of molecular weight, monosaccharide composition, and structural features. LDP-A1 (2.27 × 10^6^ Da) exhibits a significantly higher molecular weight compared to LDP-B1 (9.80 × 10^5^ Da), with distinct differences in monosaccharide types and content. Both polysaccharides contain β-glycosidic bonds. LDP-B1 adopts a sheet-like structure with an amorphous internal arrangement and a triple-helix configuration, whereas LDP-A1 is rod-shaped, with a crystalline internal structure, and lacks the triple-helix configuration. In terms of biological activity, both polysaccharides exhibit certain activities, but LDP-B1 shows significantly stronger activity in antioxidant, hypoglycemic, anti-inflammatory, and anticancer effects. In summary, LDPs exhibit significant biological activity, especially outstanding performance in antioxidant, hypoglycemic, anti-inflammatory, and anticancer effects, proving their potential for development in functional foods and pharmaceuticals. Their unique structural characteristics and diverse biological activities provide a solid theoretical foundation for further exploration of LDPs in health promotion and disease prevention, opening up new research directions and application prospects.

## 1. Introduction

*Lyophyllum decastes*, commonly referred to as the fried chicken or antler mushroom, is a rare edible fungus belonging to the Basidiomycetes class, Agaricales order, and Tricholomaceae family [1,2]. Renowned for its unique taste, texture, and nutrient-rich composition, including polysaccharides and essential amino acids [3], *L. decastes* is highly regarded as a culinary delicacy, particularly in Asia. Its potential for large-scale commercial cultivation and the growing demand for this mushroom underscore its significant economic value, further enhancing its role in both the food industry and the broader market [4]. Beyond its culinary value, this species has attracted considerable scientific interest due to its wide-ranging bioactive properties, particularly the therapeutic potential of its polysaccharides. These compounds have demonstrated antioxidant [2], antimicrobial [5], anti-inflammatory [6], and antidiabetic effects, in addition to emerging roles in metabolic regulation, such as modulating obesity [1].

There are various methods for extracting polysaccharides from edible fungi, and the choice of extraction technique can significantly affect the separation efficiency, molecular characteristics, functional properties, and biological activity of the polysaccharides [7,8]. Hot water extraction is a traditional and widely used method; however, due to its high temperature and prolonged processing time, it may lead to the degradation of sensitive components, thereby compromising the biological activity of the polysaccharides. The high temperature during the extraction process can result in the breakdown of the polysaccharide molecular structure, such as the hydrolysis of glycosidic bonds, leading to a loss of molecular weight and functional groups that are crucial for biological activity. Furthermore, heat treatment can induce conformational changes in the polysaccharide structure, potentially reducing the accessibility of active sites or compromising the overall stability of the polysaccharide. These changes may indirectly affect the interaction of polysaccharides with biological systems, thus altering their biological activity [9,10]. In recent years, emerging techniques such as ultrasound-assisted extraction, microwave-assisted extraction, and supercritical fluid extraction have gained increasing attention for improving extraction efficiency and functionality [11]. Among these, ultrasound-assisted extraction has emerged as an ideal choice due to its high efficiency and low energy consumption. The structure of edible fungus polysaccharides is closely linked to their biological activity, and an in-depth analysis of their molecular structure can elucidate their biological functions. Analyzing the sugar chain structure, molecular weight, sugar unit composition, and functional groups provides a theoretical basis for evaluating their biological activities, such as antioxidant, immunoregulatory, and anti-tumor effects [12]. The structural characteristics of polysaccharides directly influence their biological functions, and precise structural analysis forms the foundation for exploring their potential applications [13].

However, despite significant progress in ultrasound-assisted extraction technology in the field of polysaccharide extraction, research on *L. decastes* polysaccharides (LDPs) still primarily relies on hot water extraction, with relatively few studies on polysaccharide separation, purification, and biological activity assessment using ultrasound-assisted extraction technology, and no systematic research has been established. Given the close relationship between the structure and function of polysaccharides, a thorough investigation of their molecular structure and biological functions is essential for uncovering their potential biological activity [14,15]. Therefore, this study aims to systematically extract, separate, purify, and analyze the structure of LDPs using ultrasound-assisted extraction technology. Meanwhile, we will evaluate their in vitro biological activity, aiming to provide scientific evidence and experimental support for their application in fields such as food and pharmaceuticals. Through this systematic study, we hope to fill the existing research gap in the field of LDPs, provide new perspectives for developing more efficient extraction methods and a deeper understanding of the biological functions of LDPs, and promote their practical application in related industries.

## 2. Results and Discussion

### 2.1. Isolation and Purification of Polysaccharides

**Figure 1:**
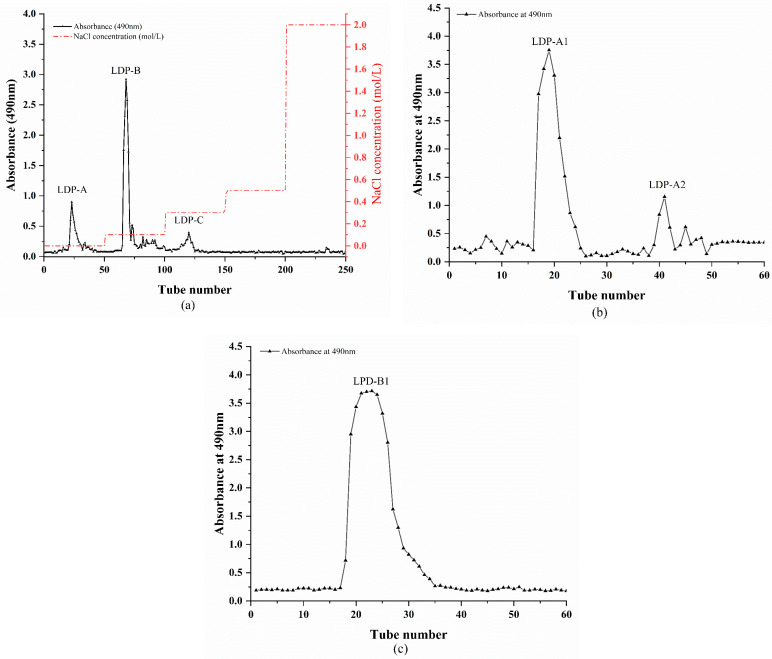
(**a**) Gradient elution curve of LDPs on DE-52 ion exchange column. (**b**) Purification curve of LDP-A on Sephacryl S-500 gel column. (**c**) Purification curve of LDP-B on Sephacryl S-500 gel column.

As shown in Figure 1a, LDPs were sequentially eluted with ultrapure water and NaCl solutions at concentrations of 0.1 M, 0.2 M, 0.3 M, 0.4 M, and 0.5 M, resulting in three polysaccharide components: LDP-A, LDP-B, and LDP-C. Of these, LDP-A, obtained by elution with ultrapure water, is a neutral polysaccharide, whereas LDP-B and LDP-C, obtained by elution with 0.1 M and 0.2 M NaCl solutions, are acidic polysaccharides. Due to the relatively low content of LDP-C, only LDP-A and LDP-B were collected, concentrated, dialyzed, freeze-dried, and stored in a dried form for future use.

As shown in Figure 1b,c, LDP-A and LDP-B were purified using a Sephacryl S-500 gel filtration column. The neutral polysaccharide LDP-A primarily consists of two components with different molecular weights, i.e., LDP-A1 and LDP-A2, while LDP-B mainly consists of a single component, LDP-B1. The three polysaccharide components (LDP-A1, LDP-A2, and LDP-B1) obtained from Sephacryl S-500 gel column separation were collected, concentrated, dialyzed, freeze-dried, and stored in a dried form for subsequent structural analysis. However, due to the relatively low yield of LDP-A2, further structural analysis of this component was not performed in this study.

### 2.2. Chemical Composition of Different LDP Components

The results of the basic chemical composition determination of the purified polysaccharide components LDP-A1 and LDP-B1 are shown in Table 1. The total sugar content of both components exceeds 85%, indicating significant purification after separation. Additionally, no protein content was detected in either polysaccharide fraction, further confirming the ideal separation and purification effect of LDPs.

### 2.3. UV Spectrum Analysis of Different LDP Components

**Figure 2:**
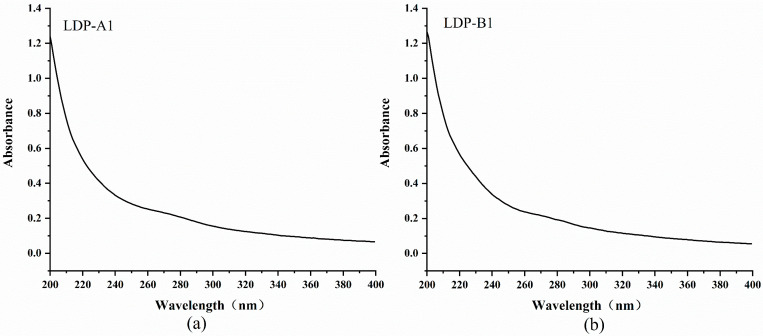
(**a**) UV spectrum of LDP-A1. (**b**) UV spectrum of LDP-B1.

As shown in Figure 2, the solutions of polysaccharide components LDP-A1 and LDP-B1 did not exhibit significant UV absorption peaks at 260 nm and 280 nm after UV scanning, indicating that these two polysaccharide components have high purity after separation and purification and contain negligible nucleic acids or proteins. This is consistent with the results of the basic chemical composition analysis (N.D.), suggesting that proteins have been almost completely removed from LDPs after separation and purification [16].

### 2.4. Monosaccharide Composition and Molecular Weight Analysis

**Figure 3:**
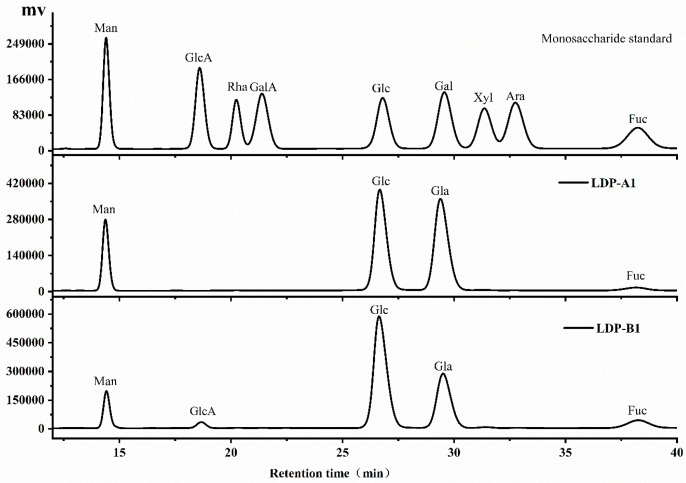
Monosaccharide compositions of LDP-A1 and LDP-B1, determined by high-performance liquid chromatography (HPLC).

As shown in Figure 3 and Table 2, the neutral polysaccharide LDP-A, further purified by Sephacryl S-500, yielded LDP-A1, which has a simpler monosaccharide composition, mainly composed of glucose and galactose, with smaller amounts of mannose and fucose. It does not contain uronic acids, further indicating that it is a neutral polysaccharide. The acidic polysaccharide LDP-B, purified by Sephacryl S-500, yielded LDP-B1, which is mainly composed of mannose, glucuronic acid, rhamnose, galacturonic acid, glucose, galactose, xylose, arabinose, and fucose. It contains a small amount of glucuronic acid, further confirming it as an acidic polysaccharide. It can be preliminarily speculated that the carbon backbone of LDP-A1 is primarily composed of glucose and galactose, with small amounts of mannose and fucose potentially interspersed in the main chain or existing as branches. The sugar chain of LDP-B1 is similar to that of LDP-A1, mainly consisting of glucose and galactose, with traces amounts of glucuronic acid and galacturonic acid, which may be interspersed in the main chain or distributed in the branches.

The monosaccharide composition results of LDPs obtained in this study are different from those reported in the relevant literature. Li et al. [3]. used hot water extraction to purify acidic polysaccharides from *L. decastes* and showed that their monosaccharide composition was mainly composed of glucose. Zhang et al. [2] obtained polysaccharides by boiling water extraction, and the monosaccharide composition was mainly composed of glucose and galactose, with small amounts of fucose, arabinose, xylose, and mannose. The molar ratio was 1.56:1.00:0.28:0.12:0.18:0.28. Zhang et al. [1]. determined the monosaccharide composition of *L. decastes* polysaccharides by hot water extraction, which consisted of glucose (82.1%), fructose (14.2%), galactose (2.5%), mannose (0.8%), fucose (0.2%), arabinose (0.1%), and rhamnose (0.04%). The main reasons for these differences could be the effects of different extraction methods, as well as the varying origins and varieties of *L. decastes*.

The ultrasonic-assisted extraction method employed in this study differs from those reported in the literature. Ultrasonic-assisted extraction utilizes intense mechanical action to disrupt cell walls and facilitate the release of polysaccharides, but it may also cause cleavage and degradation of polysaccharide chains, which can alter the monosaccharide composition and molecular weight distribution [17]. In contrast, hot water extraction relies on high temperatures and prolonged extraction times, which may lead to the hydrolysis or polymerization of certain sugars, thereby affecting the monosaccharide composition. The differences in extraction methods could be a key factor contributing to the discrepancies between our results and those reported in the literature. Additionally, the polysaccharide composition of *L. decastes* may be significantly influenced by its source, cultivation environment, and variety. Variations in growth conditions and genetic traits among *L. decastes* from different regions and varieties can lead to differences in polysaccharide composition. Previous studies have shown that the same plant species may accumulate different types of polysaccharides or exhibit variations in structural features depending on the growing environment [18]. Therefore, polysaccharides extracted from *L. decastes* from different sources and varieties may exhibit significant differences in monosaccharide composition and ratios.

**Figure 4:**
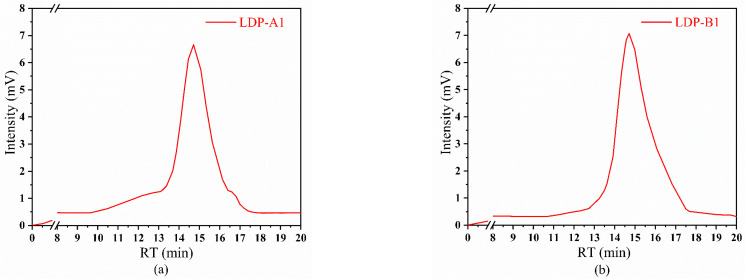
(**a**) Molecular weight of LDP-A1 and (**b**) molecular weight of LDP-B1, determined by high-performance liquid chromatography (HPLC).

From Figure 4, it can be observed that the elution peaks of LDP-A1 and LDP-B1 are single peaks, indicating that their molecular weights are relatively concentrated and that the purities of the polysaccharide components are high. The heavy average molecular weights (Mw) of LDP-A1 and LDP-B1, calculated by the assay software (Empower 3, Waters, Milford, MA, USA) based on the dextran molecular weight standard curve, were determined to be 2.27 × 10^6^ Da and 9.80 × 10^5^ Da, respectively.

This result is similar to the findings of Wang et al. [19], who isolated and purified a neutral polysaccharide (ACPN-1a) and two acidic polysaccharides (ACPA-1a and ACPA-2a) from *Auricularia auricula*. The average molecular weight of the neutral polysaccharide ACPN-1a is approximately 2.18 × 10^6^ Da, while the average molecular weight of the acidic polysaccharide ACPA-2a is 8.5 × 10^5^ Da. Both LDP-A1 and LDP-B1 components fall within the separation range of Sephacryl S-500, further validating the correctness of the choice of the dextran gel model.

### 2.5. Fourier-Transform Infrared (FTIR) Spectroscopy Analysis

**Figure 5:**
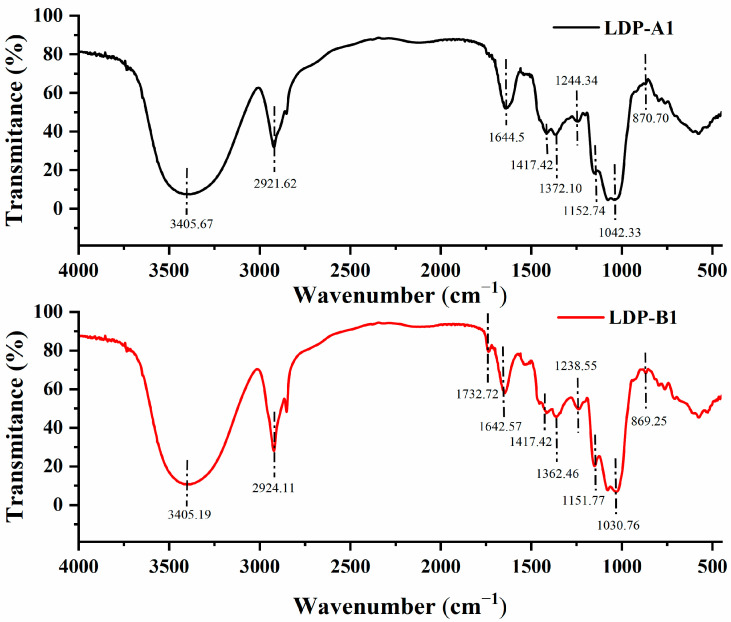
Fourier-transform infrared (FTIR) spectra of LDP-A1 and LDP-B1.

As shown in Figure 5, the infrared spectra of LDP-A1 and LDP-B1 both display the typical characteristic absorption peaks of polysaccharides, with differences in the absorption peak wavelengths between the components, indicating differences in their functional group composition and structure. The typical polysaccharide absorption peaks appear at 3405.67 cm^−1^ (LDP-A1) and 3405.19 cm^−1^ (LDP-B1), corresponding to the stretching vibration of the O-H bonds in the polysaccharide functional groups [20]. The characteristic absorption peaks at 2921.61 cm^−1^ (LDP-A1) and 2921.11 cm^−1^ (LDP-B1) correspond to the stretching vibration of the C-H bonds in the polysaccharide functional groups [21]. The absorption peaks at 1644.5 cm^−1^ (LDP-A1) and 1642.57 cm^−1^ (LDP-B1) may correspond to the bending vibration of bound water or the stretching vibration peaks of carbonyl or carbon–nitrogen double bonds (such as C=O and C=N stretching vibrations in amide bonds) [22]. The absorption peaks at 1732.72 cm^−1^ (LDP-B1) and 1249.9 cm^−1^ (LDP-B1) correspond to the stretching vibrations of C=O and C-O in ester or carboxyl groups, indicating that LDP-B1 contains uronic acid [23]. LDP-A1, however, shows no absorption peak near 1730 cm^−1^, indicating that LDP-A1 does not contain uronic acid [24]. This result is consistent with the previous monosaccharide composition analysis, further confirming that LDP-A1 is a neutral polysaccharide component, while LDP-B1 is an acidic polysaccharide component. The absorption peaks in the 1300–1475 cm^−1^ range represent C-H vibrations, indicating the deformation vibrations of the -CH_2_ functional group [25,26]. The absorption peaks in the 1000–1200 cm^−1^ range are assigned to the stretching vibrations of C-O-H or C-O-C, which are characteristic of the pyranose backbone of polysaccharides and may also include furanose rings [27]. The absorption peaks at 870.70 cm^−1^ (LDP-A1) and 869.25 cm^−1^ (LDP-B1) suggest that both polysaccharide components may contain β-configured glycosidic bonds [28].

### 2.6. Triple-Helix Determination

**Figure 6:**
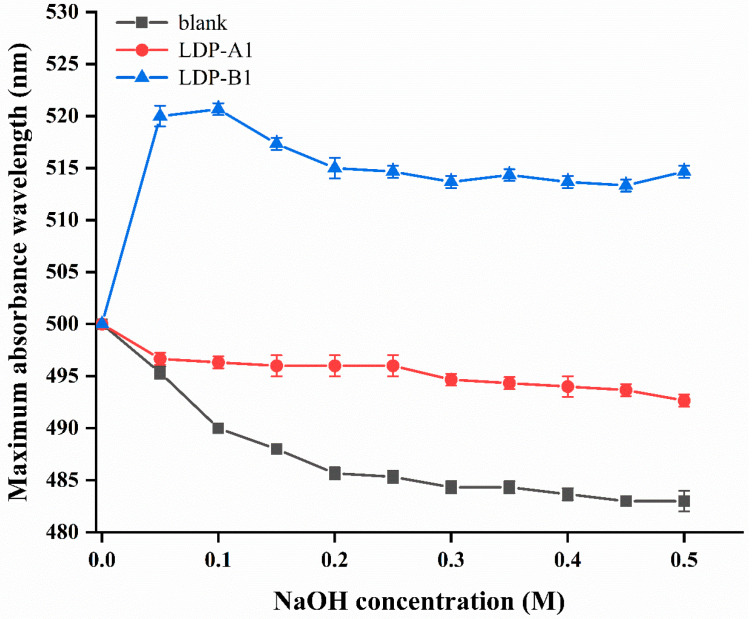
Analysis of the triple-helix structure of LDP-A1 and LDP-B1.

According to the results shown in Figure 6, when the NaOH concentration is zero, there is no significant change in the absorbance of the Congo red negative control group and the polysaccharide–Congo red solution, indicating that LDP-A1 and LDP-B1 have not formed complexes with Congo red at this point. As the NaOH concentration increases, the LDP-B1–Congo red solution undergoes a redshift, and with further increase in sodium hydroxide concentration, the absorption peak gradually shifts to the blue region. This is because at lower alkaline concentrations, after Congo red complexes with LDP-B1, the maximum absorption wavelength (λmax) of the solution undergoes a redshift, usually occurring in the range of 400 nm to 600 nm [29]. However, at higher concentrations of alkaline solution, the introduction of a higher density of charges in the helical structure causes electrostatic repulsion between the sugar chains constituting the helix, ultimately leading to the dissociation of the triple-helix structure, which causes a sudden decrease in the λmax of the LDP-B1–Congo red solution. Under different NaOH concentrations, the maximum absorption wavelength of the LDP-B1–Congo red solution changes by more than 20 nm compared to the Congo red solution [30], indicating that LDP-B1 can form an ordered triple-helix structure under weak alkaline conditions. In contrast, LDP-A1 did not show a redshift, indicating that it does not possess a triple-helix conformation. The difference in this result may be related to factors such as the monosaccharide composition, ratio, molecular weight, and glycosidic bond configuration of the two components. Based on the infrared spectroscopy results, LDP-B1 contains a large number of intramolecular and intermolecular hydrogen bonds, which help the polysaccharide form a helical structure. The interaction between glycosidic bonds and intramolecular hydrogen bonds enables the polysaccharide chains to bind to each other. In contrast, the hydrogen bonds and interactions between solvent molecules and polysaccharides further promote the formation of the triple-helix structure [31]. Polysaccharides with a triple-helix structure can form complexes with Congo red in a weak alkaline environment, stabilized by hydrogen bonds and hydrophobic interactions [30]. In contrast, other conformations do not show spectral changes. Overall, the spatial conformation of the higher-order structure has a more significant impact on the activity of polysaccharides. Specific spatial conformations are the basis for polysaccharides to exert relevant biological activities, with the triple-helix structure being the most active conformation.

### 2.7. Scanning Electron Microscopy (SEM)

**Figure 7:**
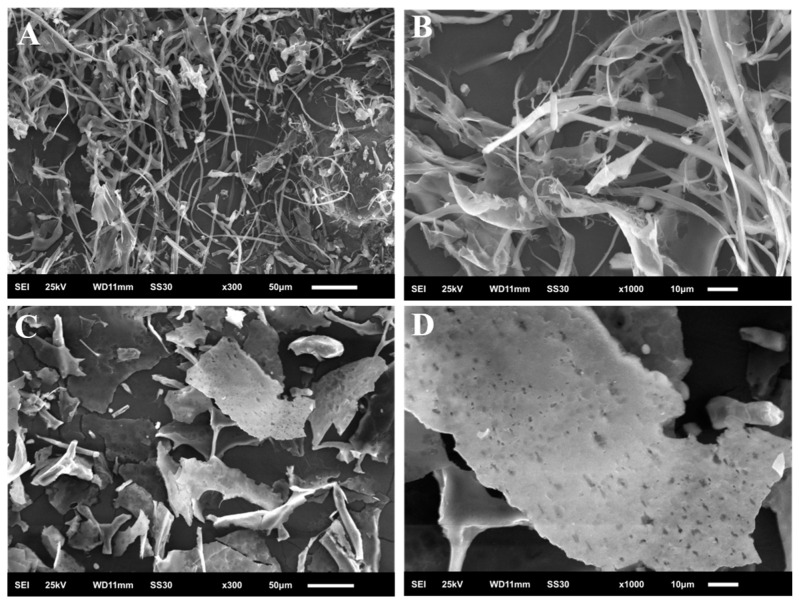
(**A**) LDP-A1 (300×); (**B**) LDP-A1 (1000×); (**C**) LDP-B1 (300×); and (**D**) LDP-B1 (1000×).

According to the observations shown in Figure 7, LDP-A1 and LDP-B1 exhibit significant differences in their microscopic structures. Under a magnification of 300×, LDP-A1 shows irregular filamentous and fibrous strip forms, displaying a chain-like conformation. This structure may result in higher viscosity for LDP-A1 [32,33]. The filamentous morphological characteristics enable the polysaccharide particles to have a larger surface area, which increases the contact area with water molecules, thereby improving solubility [34]. Furthermore, the beaded structure at the ends of the fibers, characterized by a large surface area and abundant surface pores, facilitates the effective encapsulation of drug molecules, enhances the drug-loading capacity, and allows for controlled release through the pores. This indicates that LDP-A1 has significant potential for use in drug delivery systems and can achieve encapsulation functionality [35]. Compared to LDP-A1, the surface microstructure of the acidic polysaccharide LDP-B1 is plate-like, with a few small particles and rod-like structures, which may be due to the lower molecular weight of the acidic polysaccharide [36]. At higher magnifications, the plate-like, rod-like, and small particle structures of LDP-B1 clearly show irregular cavities on their surfaces, which are likely caused by the cavitation effect during ultrasonic treatment. Ultrasonic treatment not only promoted the cleavage of polysaccharide aggregates but also likely formed new polysaccharide aggregates due to the high-energy impact of prolonged cavitation [37]. In summary, LDP-A1 and LDP-B1 show significant differences in surface morphology, structural features, and aggregate characteristics. These differences may be closely related to their molecular weights, monosaccharide compositions, and glycosidic bond structures, and they may have varying effects on their solubility, viscosity, and biological activity.

### 2.8. X-Ray Diffraction (XRD) Analysis

**Figure 8:**
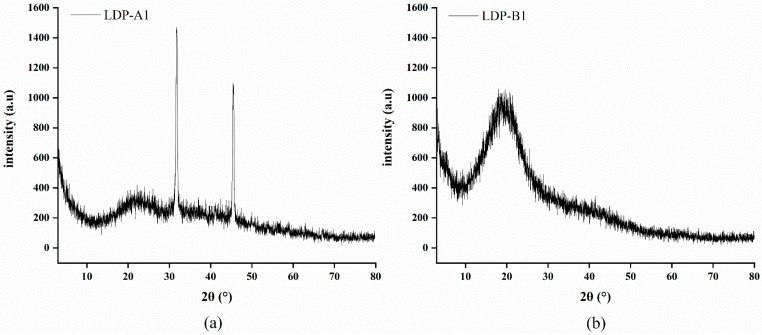
(**a**) XRD of LDP-A1. (**b**) XRD of LDP-B1.

As shown in Figure 8, within the 2θ range of 10–30°, both LDP-A1 and LDP-B1 show a broad diffraction peak around 2θ = 20°, which corresponds to the Type II crystal structure [38]. LDP-B1 exhibits only one broad peak in the crystallization reflection region, while the other peaks are weaker, indicating that it may have a crystalline structure, but the crystallization effect is not obvious. Therefore, LDP-B1 is considered an amorphous polysaccharide structure [39]. LDP-A1 shows a sharp peak around 32° and a distinct sharp peak near 45°, indicating that LDP-A1 is more likely to be in a semi-crystalline state, with the central crystallization reflection at 32° [40]. The above results are similar to those obtained by Zhang et al. [41] in their research on perilla polysaccharides and by Ren et al. [42] in their quinoa polysaccharide study. Compared to LDP-B1, LDP-A1 has a significantly higher degree of crystallinity, with some distinct sharp crystal peaks within the measurement range, which small molecular sugar substances may cause. This phenomenon in polysaccharides may have occurred due to changes in the crystal structure during the early purification process, causing the polysaccharide to transition from an amorphous state to a semi-crystalline state [42].

### 2.9. Antioxidant Activity Analysis

#### 2.9.1. ABTS Radical Scavenging Activity

**Figure 9:**
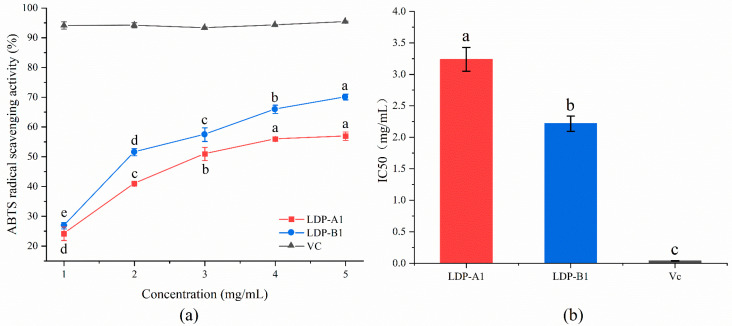
(**a**) ABTS radical scavenging ability. (**b**) IC50. Note: the use of different letters denotes significant differences between the corresponding values (*p* < 0.05).

As shown in Figure 9, the scavenging rate of ABTS free radicals by LDP-A1 and LDP-B1 increased in a dose-dependent manner with the sample concentration. When the sample concentration reached 5 mg/mL, the scavenging rates of LDP-A1 and LDP-B1 were 56.90 ± 1.41% and 70.07 ± 0.95%, respectively, with IC50 values of 3.24 ± 0.19 and 2.22 ± 0.12 mg/mL. IC50 (the half-maximal inhibitory concentration) is the concentration of a substance that is required to inhibit a specific biological or biochemical function by 50%. The overall trend of change for LDP-A1 and LDP-B1 was similar, but LDP-B1 exhibited stronger ABTS scavenging ability than LDP-A1. Furthermore, after the concentration reached 5 mg/mL, the scavenging rates of both stabilized but remained much lower than the scavenging capacity of vitamin C (Vc) (95.43 ± 0.21%).

This difference may be related to the triple-helix conformation of LDP-B1. The triple-helix structure of LDP-B1 helps form a more stable structure for the sugar molecules, which may enhance its biocompatibility and antioxidant activity. The triple-helix structure allows the sugar molecules to exist in a more ordered manner in aqueous solution, thereby optimizing their reaction efficiency with free radicals. In contrast, LDP-A1, which does not have the triple-helix structure and has a larger molecular weight, may result in less efficient organization of its sugar molecules in solution, potentially reducing its effectiveness in scavenging free radicals. At the same time, the helical structure of LDP-B1 may expose more active sites, such as carboxyl groups and other electron-withdrawing groups, which aid in free radical scavenging. In conclusion, the molecular structure and characteristics of LDP-B1 enable it to exhibit more potent antioxidant activity in scavenging ABTS free radicals [31].

#### 2.9.2. DPPH Radical Scavenging Activity

**Figure 10:**
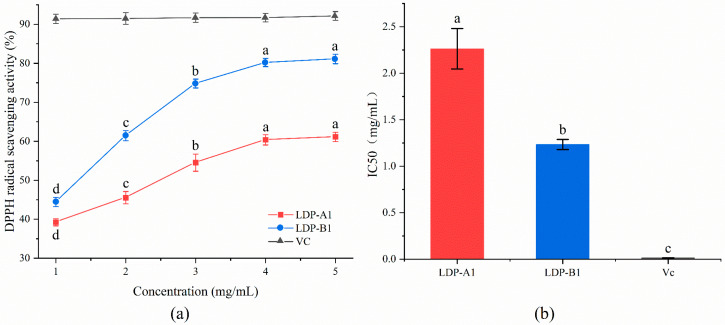
(**a**) DPPH radical scavenging ability. (**b**) IC50. Note: the use of different letters denotes significant differences between the corresponding values (*p* < 0.05).

As shown in Figure 10, with the increase in sample concentration, the scavenging ability of LDP-A1 and LDP-B1 against DPPH free radicals gradually strengthened. At a sample concentration of 1 mg/mL, the scavenging rates of LDP-A1 and LDP-B1 were 39.23 ± 0.92% and 44.42 ± 1.21%, respectively, which were significantly lower than the scavenging rate of Vc (91.39 ± 1.14%) (*p* < 0.01). However, when the concentration increased to 5 mg/mL, the scavenging rate of LDP-B1 reached 81.12 ± 1.24%, close to that of Vc (92.13 ± 1.12%). In addition, the IC50 values of LDP-A1, LDP-B1, and Vc were 2.26 ± 0.22, 1.24 ± 0.05, and 0.014 ± 0.002 mg/mL, respectively, indicating that LDP-B1 has a stronger scavenging ability at the same concentration. This may be related to LDP-B1 being an acidic sugar that contains a higher content of uronic acid and sulfate groups, which is consistent with the research of Song et al. [43]. It may also be because LDP-B1 has the lowest average molecular weight, and at the same concentration, it contains more hydroxyl functional groups. Additionally, the charged groups in acidic sugars (such as carboxyl and sulfate groups) typically have higher water solubility and hydrophilicity, enabling them to react more effectively with DPPH free radicals in an aqueous solution. In contrast, neutral sugars have poorer hydrophilicity, and in aqueous solutions, they may not exhibit an antioxidant capacity as strong as that of acidic sugars [44].

#### 2.9.3. Hydroxyl Radical Scavenging Activity

**Figure 11:**
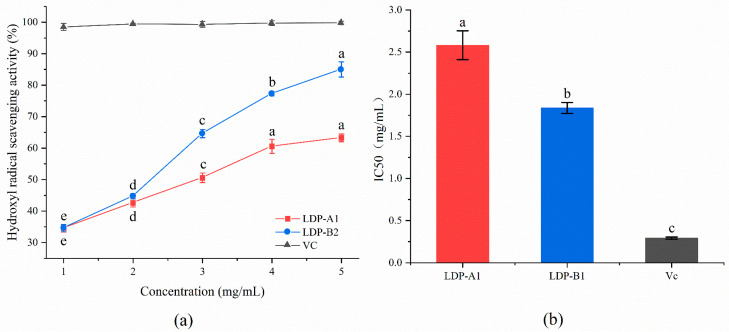
(**a**) Hydroxyl radical scavenging ability. (**b**) IC50. Note: the use of different letters denotes significant differences between the corresponding values (*p* < 0.05).

As shown in Figure 11, within the selected concentration range, LDP-A1 and LDP-B1 demonstrate a good scavenging effect on hydroxyl radicals. As the concentration increases, the scavenging effect gradually strengthens, showing a certain dose–effect relationship. When the sample concentration is 5 mg/mL, the scavenging rates of LDP-A1 and LDP-B1 are 63.26 ± 1.23% and 85.01 ± 2.41%, respectively, with IC50 values of 2.58 ± 0.17 mg/mL and 1.84 ± 0.07 mg/mL, both higher than the positive control Vc (0.29 ± 0.02 mg/mL). Among them, LDP-B1 exhibits a relatively stronger scavenging effect. This phenomenon may be related to the content of uronic acid in LDP-B1. The carboxyl group of uronic acid can react with ·OH radicals through electron donation, thereby scavenging the free radicals. Research has shown that polysaccharide samples with higher uronic acid content have stronger free radical scavenging ability [45]. In addition, acidic sugars may either directly react with ·OH to form stable products or indirectly enhance the activity of antioxidant enzymes, thereby improving the overall antioxidant capacity. The combined action of these mechanisms results in acidic sugars exhibiting stronger activity in scavenging ·OH radicals. Therefore, LDP-B1 demonstrates stronger antioxidant effects compared to LDP-A1.

#### 2.9.4. Reducing Power

**Figure 12:**
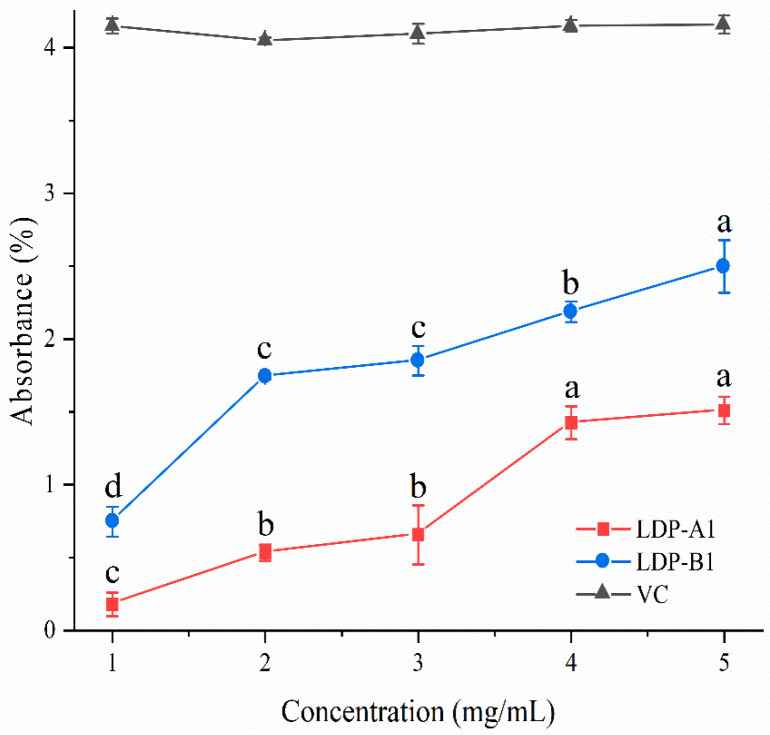
Reducing power. Note: the use of different letters denotes significant differences between the corresponding values (*p* < 0.05).

As shown in Figure 12, the reducing abilities of LDP-A1 and LDP-B1 are dose-dependent, and both are lower than the reducing ability of Vc. The reducing abilities of LDP-A1 and LDP-B1 change more gradually with varying sample concentrations, but overall, they are lower than that of Vc, which may be related to their polyphenol content and molecular size. While we did not directly measure the polyphenol content in the fractions, it is well established in the literature that polyphenols play a significant role in reducing ability. Studies have shown that smaller molecular sizes help expose more reducing ends, thereby providing more active sites (such as carboxyl groups, amino groups, etc.). These reaction sites can interact with metal ions or free radicals in reduction reactions, improving reduction efficiency [46]. Therefore, the reducing abilities of LDP-A1 and LDP-B1 are weaker compared to Vc, possibly due to fewer or less accessible active sites in their molecular structures, making it harder for them to participate in reduction reactions.

### 2.10. Hypoglycemic Activity Analysis

#### 2.10.1. α-Amylase Inhibitory Activity

**Figure 13:**
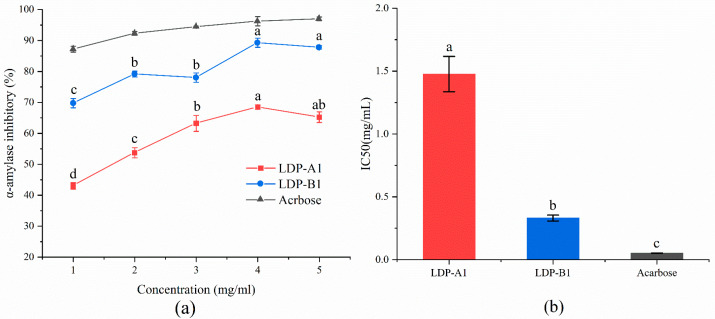
(**a**) α-amylase inhibition ability. (**b**) IC50. Note: the use of different letters denotes significant differences between the corresponding values (*p* < 0.05).

Figure 13 shows the inhibitory effects of LDP-A1, LDP-B1, and acarbose on α-amylase within the concentration range of 1–5 mg/mL, exhibiting a significant dose–effect relationship. As the concentration increases, the inhibitory ability of both polysaccharides on α-amylase gradually strengthens, but the overall inhibition effect is lower than that of acarbose, with LDP-B1 showing a stronger inhibitory ability than LDP-A1, which may be related to the higher uronic acid content in LDP-B1 [47]. When the concentration reaches 4 mg/mL, the inhibition rates of LDP-A1, LDP-B1, and acarbose are 68.49 ± 0.75%, 89.29 ± 1.51%, and 96.25 ± 1.46%, respectively, with corresponding IC50 values of 1.48 ± 0.14, 0.33 ± 0.02, and 0.052 ± 0.001 mg/mL. After increasing the polysaccharide concentration to 5 mg/mL, the inhibition rates of both polysaccharides slightly decrease, possibly due to enhanced interactions between polysaccharide molecules at high concentrations (such as hydrogen bonding or hydrophobic interactions), leading to the formation of small aggregates, which block some active sites and reduce the binding efficiency with the enzyme. In addition, the viscosity of the solution significantly increases, leading to a reduction in flowability and molecular diffusion rate, which weakens the inhibition effect [48]. Overall, the inhibition rate of LDP-A1 on α-amylase does not exceed 70%, indicating that the inhibitory effect of LDP-A1 on α-amylase is relatively limited.

#### 2.10.2. α-Glucosidase Inhibitory Activity

**Figure 14:**
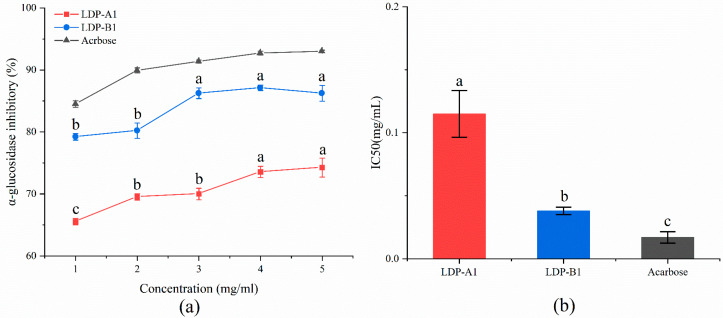
(**a**) α-glucosidase inhibition ability. (**b**) IC50. Note: the use of different letters denotes significant differences between the corresponding values (*p* < 0.05).

As shown in Figure 14, LDP-A1 and LDP-B1 exhibit significant dose-dependent inhibition of α-glucosidase. As the polysaccharide concentration increases from 0 to 5 mg/mL, the inhibitory effects of LDP-A1, LDP-B1, and acarbose on α-glucosidase gradually enhance, with inhibition rates of 74.25 ± 1.52%, 86.25 ± 1.27%, and 93.01 ± 0.21%, respectively, and corresponding IC50 values of 0.115 ± 0.019, 0.038 ± 0.003, and 0.017 ± 0.045 mg/mL. Compared to acarbose, both polysaccharides show slightly lower inhibitory abilities, with LDP-B1 demonstrating better inhibition than LDP-A1. This difference may be related to their uronic acid content and α-(1→4) glycosidic bond structure [49]. The presence of uronic acid and α-(1→4) glycosidic bonds may enhance the affinity of the polysaccharides for α-glucosidase, making it easier for them to bind to the enzyme’s active site, thereby inhibiting the enzyme’s activity. Specifically, the carboxyl group of uronic acid can bind to the enzyme’s positive charge site through electrostatic interaction, while the α-(1→4) glycosidic bond may interact with the enzyme’s active site, further hindering the enzyme’s substrate binding site [49,50]. When the polysaccharide concentration reaches 5 mg/mL, the inhibition rates of LDP-A1 and LDP-B1 both exceed 70%, further confirming their significant inhibitory effect on α-glucosidase. Compared to the inhibition of α-amylase, the polysaccharides show a more prominent inhibitory effect on α-glucosidase, which is consistent with the findings of Wan et al. [51] that the mechanisms of action of polysaccharides on α-glucosidase and α-amylase may differ [52].

In summary, LDPs and their acidic polysaccharide components exhibit significant hypoglycemic potential by inhibiting α-glucosidase and α-amylase activity. Previous studies have shown that strong inhibition of α-amylase often leads to side effects such as gastrointestinal discomfort [53]. According to the IC50 results, at low concentrations, LDPs can effectively inhibit α-glucosidase activity while having a milder inhibitory effect on α-amylase, thereby regulating postprandial blood glucose while minimizing side effects. Moreover, studies have shown that the hypoglycemic activity of polysaccharides is closely related to their chemical structure, including molecular weight, monosaccharide composition, and glycosidic bond types [54]. In general, polysaccharides with more complex monosaccharide compositions, particularly those with varying proportions of arabinose, galactose, glucose, xylose, and small amounts of rhamnose, usually demonstrate better hypoglycemic activity [55,56]. Based on this characteristic, LDP-B1 shows potential value as a therapeutic agent for diabetes.

### 2.11. Anti-Inflammatory Activities

#### 2.11.1. Cell Proliferation Rate

**Figure 15:**
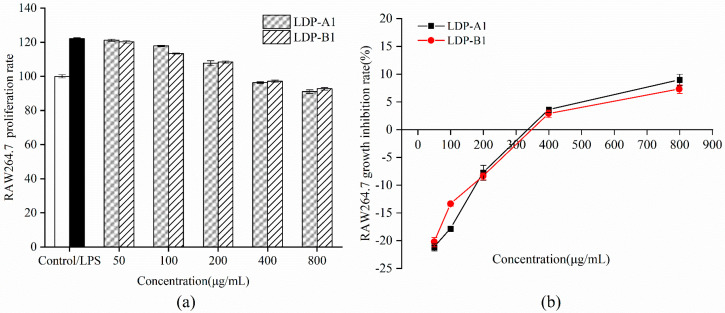
(**a**) Cell viability. (**b**) Cell growth inhibition rate.

To select the appropriate experimental concentrations for mouse macrophage RAW264.7 cells, this study incubated Lipopolysaccharide (LPS) and different concentrations of polysaccharide samples with RAW264.7 cells for 24 h, and cell growth was assessed using the CCK-8 assay. The effects of LDP-A1 and LDP-B1 on RAW264.7 cell proliferation are shown in Figure 15. LPS treatment significantly activated RAW264.7 cells, with a significant difference compared to the control group (*p* < 0.05). Compared to the LPS group, LDP-A1 and LDP-B1 showed some inhibitory effect on the proliferation of LPS-stimulated RAW264.7 cells at low concentrations, but no significant difference was observed (*p* > 0.05). At concentrations of 50 μg/mL, 100 μg/mL, and 200 μg/mL, the cell viability of both polysaccharide components was ≥100%, showing no cytotoxicity. At a concentration of 800 μg/mL, the growth inhibition rates of LDP-A1 and LDP-B1 on RAW264.7 cells were 8.95% and 7.29%, respectively, with cell viability remaining above 90%. Therefore, the experimental results indicate that LDP-A1 and LDP-B1 do not exhibit significant cytotoxicity within the selected concentration range, and concentrations of 50, 100, and 200 μg/mL can be chosen for subsequent experiments.

#### 2.11.2. Effects of Polysaccharide Samples on LPS-Induced NO and Cytokine Secretion in RAW264.7 Cells

**Figure 16:**
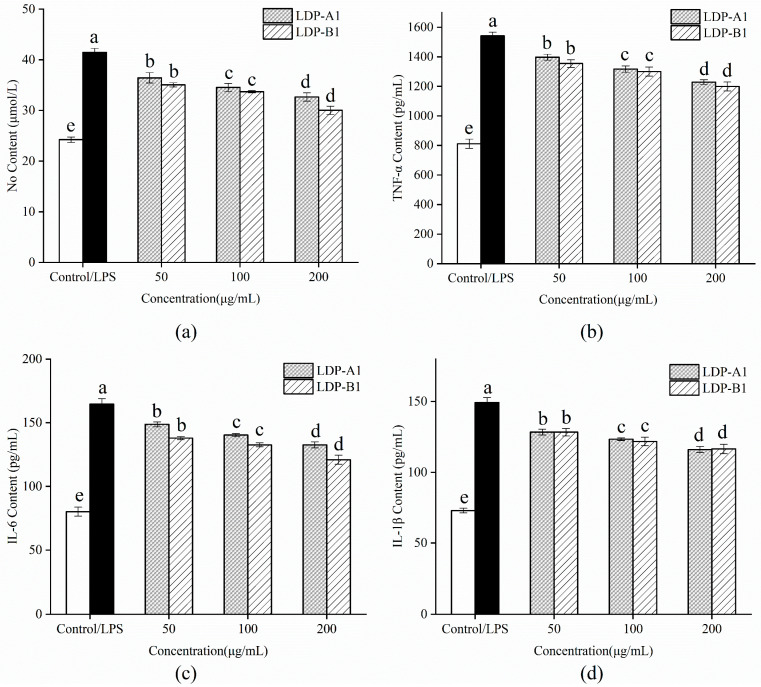
(**a**) Effect on NO production. (**b**) Effect on TNF-α production. (**c**) Effect on IL-6 production. (**d**) Effect on IL-1β production. Note: the use of different letters denotes significant differences between the corresponding values (*p* < 0.05).

As shown in Figure 16a, after stimulating RAW264.7 mouse macrophages with 1 μg/mL LPS, the NO content in the cell culture supernatant of the LPS group was significantly higher than that in the control group without LPS stimulation (*p* < 0.05), indicating that LPS stimulation successfully induced an inflammatory response model in RAW264.7 cells. The LDP-A1 and LDP-B1 treatment groups significantly inhibited the release of the signaling molecule NO induced by LPS stimulation in RAW264.7 cells, showing protective effects. Compared to the LPS group, significant inhibitory effects were observed starting at a concentration of 50 μg/mL (*p* < 0.05), and the inhibitory effect increased in a dose-dependent manner as the concentration increased, with LDP-B1 showing the most significant inhibitory effect.

As shown in Figure 16, under 1 μg/mL LPS stimulation, the levels of the inflammatory cytokines IL-6, TNF-α, and IL-1β in the cell culture supernatant of the LPS group were significantly higher than those in the control group without LPS stimulation (*p* < 0.05). After intervention with LDP-A1 and LDP-B1, the release of inflammatory cytokines IL-6, TNF-α, and IL-1β induced by LPS in RAW264.7 cells was significantly inhibited, exerting anti-inflammatory protective effects. Within the concentration range of 50~200 μg/mL, the inhibitory effect increased with concentration, and both LDP-A1 and LDP-B1 exhibited a dose-dependent inhibition of the LPS-induced production of inflammatory cytokines IL-6, TNF-α, and IL-1β. Notably, the two polysaccharide components showed minimal difference in their effect on the inflammatory cytokine IL-1β, while LDP-B1 exhibited a more pronounced inhibitory effect on IL-6 and TNF-α. Previous studies have suggested that polysaccharides inhibit inflammation through factors such as molecular weight, monosaccharide composition, glycosidic bonds, and conformation [57,58,59]. Polysaccharides with low molecular weight easily pass through the cell membrane and bind to receptors, thereby inhibiting the release of inflammatory mediators [60]. The monosaccharide composition determines the affinity of the polysaccharide for the receptors, while the glycosidic bonds and conformation affect its spatial structure, thereby regulating the activation of inflammatory pathways and reducing the release of pro-inflammatory factors. Therefore, LDP-A1 and LDP-B1 may exhibit anti-inflammatory properties, but due to their structural differences, their anti-inflammatory effects vary.

In summary, LDP-A1 and LDP-B1 are safe without cytotoxicity to mouse macrophage RAW264.7 cells within the experimental concentration range and can inhibit the substantial release of inflammatory mediators NO, IL-6, TNF-α, and IL-1β induced by LPS to varying degrees, thereby exerting anti-inflammatory and protective effects. Due to their significant anti-inflammatory effects and good dose dependency, LDP-A1 and LDP-B1 are expected to serve as potential anti-inflammatory agents, providing new ideas and evidence for the treatment of inflammation-related diseases.

### 2.12. Anti-Tumor Activity

**Figure 17:**
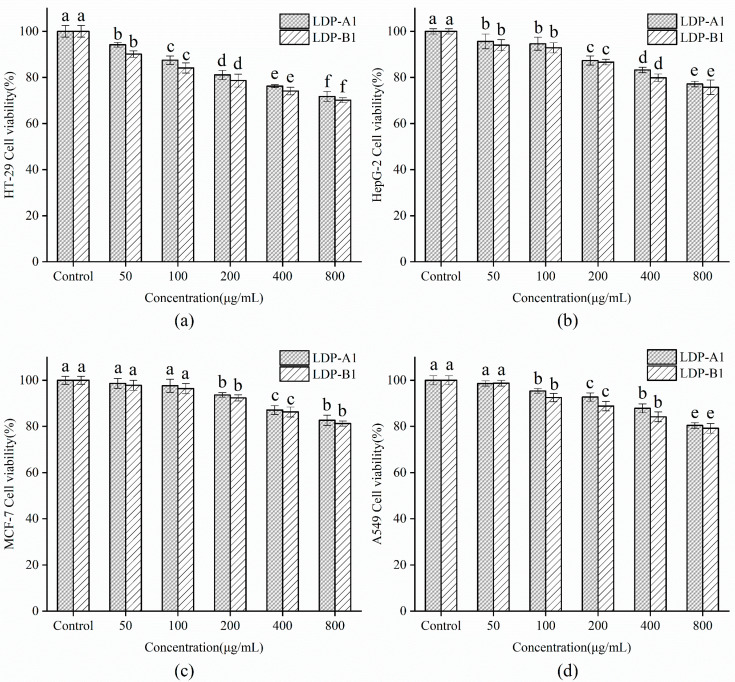
(**a**) Effect on HT-29 cell viability. (**b**) Effect on HepG-2 cell viability. (**c**) Effect on MCF-7 cell viability. (**d**) Effect on A549 cell viability. Note: the use of different letters denotes significant differences between the corresponding values (*p* < 0.05).

As shown in Figure 17, samples of LDP-A1 and LDP-B1 at different concentrations exhibit varying degrees of proliferation inhibition on tumor cells HT-29, HepG-2, MCF-7, and A549, with the inhibitory effects being dose-dependent. At a concentration of 800 μg/mL, the cell viability of the four tumor cell lines after adding LDP-A1 and LDP-B1 were 71.75 ± 2.21% and 70.15 ± 1.10% (HT-29), 77.15 ± 1.21% and 75.75 ± 3.1% (HepG-2), 82.66 ± 2.23% and 81.22 ± 1.11% (MCF-7), and 80.41 ± 1.2% and 79.17 ± 2.12% (A549), respectively. The inhibitory effects of both samples on HepG-2, MCF-7, and A549 were relatively weak, but they exhibited more significant inhibitory effects on HT-29, with LDP-B1 showing better inhibition than LDP-A1. The difference in anti-tumor activity between the two components may be related to structural characteristics such as molecular weight, branching degree, and the presence of a triple-helix configuration [61,62]. This issue needs to be further explored through experimental analysis to better understand the anti-tumor activity mechanism and structure–activity relationship. Notably, while LDP-B1 exerts a relatively stable therapeutic effect on colon cancer (HT-29 cells), it has low toxicity and few side effects on normal cells, making it highly safe. This characteristic gives LDP-B1 significant potential in the development of anti-tumor drugs, especially as a replacement for traditional chemotherapy agents, with considerable promise.

## 3. Materials and Methods

### 3.1. Materials and Reagents

*L. decastes* specimens were purchased from Weier Commerce and Trade Co., Ltd. in Gutian County (Ningde, China). DEAE cellulose-52 and Sephacryl S-500 were obtained from Ruida Henghui Technology Co., Ltd. (Beijing, China). The monosaccharide standards were purchased from Yuan Ye Biotechnology Co., Ltd. (Shanghai, China). The cell lines used in our study, including RAW 264.7 (CL-0190), HepG-2 (CL-0103), A549 (CL-0016), MCF7 (CL-0149), and HT-29 (CL-0118), were all purchased from Wuhan Pricella Life Science & Technology Co., Ltd. (Wuhan, China). In this study, all chemicals and solvents used were strictly selected analytical-grade (AR) products to ensure the accuracy and reliability of the experimental results and were supplied by Yuan Ye Biotechnology Co., Ltd. (Shanghai, China).

### 3.2. Preparation of Polysaccharides from L. decastes

Based on the existing research with slight modifications [19], the ultrasonic-assisted extraction method was used to extract crude polysaccharides from the powder of the fruiting bodies of *L. decastes*. The specific steps are as follows: first, the ultrasonic probe was inserted into the middle of the sample solution in the conical flask, positioned between the liquid surface and the bottom. Extraction was then performed according to specific ultrasonic parameters (ultrasonicate for 10 s, followed by a 5 s pause). After the ultrasonic treatment, the sample solution was transferred to a water bath for hot water extraction, with the process concluded once the extraction was complete.

The extraction conditions are as follows: ultrasonic treatment time of 30 min, ultrasonic power of 300 W, liquid-to-material ratio of 30 mL/g, extraction temperature of 70 °C, and hot water extraction time of 3 h. After extraction, steps such as alcohol precipitation, protein removal by the trichloroacetic acid method [63], decolorization by AB-8 macroporous resin [64], centrifugation, concentration, and freeze-drying were performed to obtain the dried crude polysaccharides from *L. decastes*. Subsequently, the obtained crude polysaccharides were prepared into a 5 mg/mL solution, dialyzed (with a molecular weight cutoff of 8 kDa) to remove small molecular impurities, and finally freeze-dried for later use.

### 3.3. Isolation and Purification of Polysaccharides from LDPs

First, activate the DEAE cellulose-52 anion exchange resin according to the instructions to ensure it is fully swollen and restored to its active state. Pack the column with the wet packing material (2.5 cm × 60 cm). Then, equilibrate the chromatography column with deionized water at a flow rate of 1.0 mL/min. After eluting 3–5 column volumes, begin the polysaccharide separation step. Take 2 g of LDP and dissolve it in distilled water to prepare a 20 mg/mL polysaccharide solution. After centrifuging at 5000 rpm for 20 min to remove the precipitate, filter the supernatant using a 0.45 μm microporous filter membrane to obtain the polysaccharide solution with impurities removed, ready for column application. Slowly inject the polysaccharide solution into the equilibrated separation column using a 5 mL sterile syringe, allow it to equilibrate for 10 min, and then begin elution. Sequentially elute with deionized water and 0.1 M, 0.2 M, 0.3 M, and 0.5 M NaCl solutions for two column volumes at a flow rate of 1.0 mL/min, collecting 5 mL per tube. Subsequently, the phenol–sulfuric acid method is used to detect the sugar content in the eluate and plot the elution curve. Collect and combine fractions of the same component, vacuum concentrate, dialyze against flowing water (molecular weight cutoff of 8 kDa) for 72 h, and freeze-dry. After the activation and pre-treatment of Sephacryl S-500, perform wet packing of the column (1.5 cm × 80 cm). Then, equilibrate the column with NaCl solution at a flow rate of 0.15 mL/min for 16 h. After equilibrium, add the polysaccharide fractions collected in the previous step and elute at a flow rate of 0.15 mL/min, collecting 3 mL per tube every 20 min. Plot the elution curve, collect the elution peaks, vacuum concentrate, dialyze (molecular weight cutoff of 8 kDa) for desalting, and then freeze-dry to obtain a relatively homogeneous polysaccharide fraction for later use.

### 3.4. Determination of Chemical Composition

The total sugar content was determined using the phenol–sulfuric acid method, as described in previous reports [65]. The protein content was determined using the Coomassie Brilliant Blue method, as described in earlier reports [66].

### 3.5. UV Full-Wavelength Scan Analysis

Weigh a certain amount of each purified polysaccharide sample, prepare a 0.5 mg/mL solution of the polysaccharide purified component, and scan the ultraviolet absorption spectrum of the polysaccharide solution in the wavelength range of 200–600 nm.

### 3.6. Monosaccharide Composition and Molecular Weight

Monosaccharide compositions were analyzed by high-performance liquid chromatography (HPLC) [67] by hydrolyzing 2 mg of polysaccharide samples with 1 mole/liter hydrochloric acid in anhydrous methanol for 12–16 h at 80 °C, followed by further hydrolysis using 2 mole/liter trifluoroacetic acid (TFA) for 1 h at 120 °C. The hydrolyzed products were then derivatized using 1-phenyl-3-methyl-5-pyrazolone (PMP). To begin, 500 µL of 0.3 mol/L NaOH solution was added to the dried sample and the standard, followed by thorough mixing to ensure complete dissolution of the sample. Next, 500 µL of PMP-methanol solution was added, and the mixture was well mixed before transferring 200 µL of it into a 1.50 mL EP tube. The tube was incubated at 70 °C in a water bath for 0.5 h. After incubation, 100 µL of 0.3 mol/L HCl solution was added and mixed thoroughly; then, 700 µL of CH_2_Cl_2_ was introduced. The mixture was vortexed for 90 s and centrifuged at 8000 rpm for 3 min. The CH_2_Cl_2_ layer was discarded, and the aqueous phase was retained. This extraction procedure was repeated three times. The chromatographic column was a DIKMA Inertsil ODS-3 (4.6 × 150 mm). The mobile phase consisted of a mixture of 82.0% phosphate buffer (PBS, 0.1 M, pH 7.0) and 18.0% acetonitrile (*v*/*v*) at a flow rate of 1 mL/min, maintaining a column temperature of 30 °C, with the derivatives injected in volumes of 20 μL. Monitoring occurred through UV absorption at 245 nm using an SPD-10AVD UV–Vis detector.

Molecular weight determination was conducted through high-performance liquid chromatography (HPLC) [68]. For sample preparation, 50 mg of the sample was weighed into a 10 mL volumetric flask, dissolved in the mobile phase, and made up to volume. The analytical instrument used was a Waters 2695 high-performance liquid chromatograph equipped with a 2410 Differential Refractometer Detector and Empower workstation. The chromatographic column was an Ultrahydrogel TM Linear (Waltham, MA, USA), 300 mm × 7.8 mm id. The mobile phase was 0.1 M sodium nitrate, with a flow rate of 0.5 mL/min and a column temperature of 40 °C. A calibration curve was constructed using glucose standards (T-2000, T-300, T-150, T-10, T-5, and glucose 180 China National Institute for Food and Drug Control, Beijing, China).

### 3.7. Fourier-Transform Infrared (FTIR) Spectroscopy Analysis

Approximately 2 mg of the dried polysaccharide sample was weighed, mixed thoroughly with about 300 mg of dried potassium bromide (KBr) powder, and then pressed into a pellet (1 mm thickness). Spectral measurements were performed using a Fourier-transform infrared spectrometer (Spectrum 100, PerkinElmer Co., Waltham, MA, USA) in the wavelength range of 450–4000 cm^−1^.

### 3.8. Triple-Helix Determination

The Congo red assay [69] was used to determine whether the polysaccharide sample has a triple-helix structure. Briefly, 5 mg of polysaccharide was dissolved in 2.0 mL of ultrapure water and mixed with 2 mL of Congo red solution (80 μmol/L); then, an appropriate volume of NaOH solution (1 mol/L) was added to make the NaOH concentration in the mixture (0, 0.05, 0.10, 0.15, 0.20, 0.25, 0.30, 0.35, 0.40, 0.45, and 0.50 mol/L) in increasing order. After standing at room temperature for 10 min, the characteristic absorption spectra in the range of 190–600 nm were scanned and recorded using a UV–Visible spectrophotometer.

### 3.9. Scanning Electron Microscopy (SEM)

A small amount of dried polysaccharide sample was placed on the sample holder black tape, and then gold coating treatment was performed. The microstructure was observed and recorded using a scanning electron microscope (JSM-6510LA, JEOL Ltd., Tokyo, Japan).

### 3.10. X-Ray Diffraction (XRD) Analysis

XRD testing was performed using a Rigaku Ultima 4 X-ray diffractometer (Chiyoda-ku, Tokyo, Japan), with method adjustments based on the literature [70]. The dried polysaccharide sample was placed in the sample holder for testing, with the measurement conditions set as follows: copper target (k = 1.5406 Å), tube voltage of 40 kV, scanning angle range of 3–80° (2θ), and scanning speed of 5°/min.

### 3.11. Antioxidant Activity Analysis

#### 3.11.1. ABTS Radical Scavenging Activity

The ABTS radical scavenging ability was determined using a previously reported method [71]. The absorbance of the solution was measured at 734 nm using a microplate reader (Varioskan LUX, Thermo Fisher Scientific, Waltham, MA, USA), with vitamin C (Vc) as the positive control. The scavenging rate was calculated according to the following formula:

(1) $$\mathrm{Scavenging}\;\mathrm{rate}\;(\%)=(1-\frac{A_{1}-A_{2}}{A_{0}})\times 100$$

In the formula, A_1_ is the absorbance of the sample solution after the reaction, A_2_ is the absorbance of the sample solution and water (deionized water is used to replace ABTS in the reaction with the sample solution), and A_0_ is the absorbance of the ABTS solution alone (deionized water is used to replace the polysaccharide solution in the reaction with ABTS).

#### 3.11.2. DPPH Radical Scavenging Activity

The DPPH radical scavenging ability was determined using a previously reported method [72]. The absorbance of different solutions was measured at 517 nm, with vitamin C (Vc) as the positive control. The DPPH radical scavenging rate was calculated using Formula (1).

In the formula, A_1_ is the absorbance of the sample solution after the reaction, A_2_ is the absorbance of the sample solution and water (using anhydrous methanol to replace DPPH in the reaction with the sample solution), and A_0_ is the absorbance of the DPPH solution alone (using anhydrous methanol to replace the polysaccharide solution in the reaction with DPPH).

#### 3.11.3. Hydroxyl Radical Scavenging Activity

The hydroxyl radical scavenging ability was determined using a previously reported method [17]. The absorbance of different solutions was measured at 510 nm, with vitamin C (Vc) as the positive control. The hydroxyl radical scavenging rate was calculated using Formula (1).

In the formula, A_1_ is the absorbance of the sample solution after the reaction, A_2_ is the absorbance of the sample solution after the reaction with deionized water replacing H_2_O_2_, and A_0_ is the absorbance of the reaction after replacing the polysaccharide solution with deionized water.

#### 3.11.4. Reducing Power

The reducing power was determined using a previously reported method [73]. The absorbance of different solutions was measured at 700 nm, with vitamin C (Vc) as the positive control, and the reducing power was calculated using Formula (2).

(2) $$\mathrm{Reduing}\;\mathrm{power}\;=A_{1}-A_{2}$$

In the formula, A_1_ is the absorbance of the sample solution after the reaction and A_2_ is the absorbance after reacting with deionized water instead of the sample.

### 3.12. Hypoglycemic Activity Analysis

#### 3.12.1. Inhibition Rate of α-Amylase

The method was slightly modified from a previously reported procedure [74]: 500 μL of polysaccharides at different concentrations were mixed with 500 μL of 1 U/mL α-amylase solution and incubated in a 37 °C water bath for 10 min. Then, 500 μL of 1% starch solution was added and incubated for another 10 min. Briefly, 1 mL of DNS solution was added to terminate the reaction, heated for 5 min, and then cooled and diluted. The absorbance was measured at 540 nm using a spectrophotometer, and the inhibition rate was calculated according to Formula (3), with acarbose as the control.

(3) $$\mathrm{Inhibitory}\;\mathrm{rate}\;(\%)=(1-\frac{A_{1}-A_{2}}{A_{0}})\times 100$$

In the formula, A_1_ is the absorbance of the reaction system after adding the polysaccharide sample solution, A_0_ is the absorbance measured when PBS is used as a substitute for the sample solution, and A_2_ is the absorbance measured when PBS is used as a substitute for α-amylase.

#### 3.12.2. Inhibition Rate of α-Glucosidase

A slightly modified version of a previously reported method [75] was used: 50 μL of polysaccharide solution at different concentrations was mixed with 50 μL of 0.5 U/mL α-glucosidase solution and incubated at 37 °C for 10 min. Then, 100 μL of 5 mmol/L PNPG solution was added, mixed to start the reaction, and incubated for another 30 min. After the reaction, 100 μL of 1 mol/L sodium carbonate solution was added to stop the reaction, and the mixture was cooled to room temperature. The absorbance was measured at 405 nm using a spectrophotometer, and the inhibition rate was calculated using Formula (3), with acarbose as a positive control.

In the formula, A_1_ is the absorbance of the reaction system after adding the polysaccharide sample solution, A_0_ is the absorbance measured when PBS is used as a substitute for the sample solution, and A_2_ is the absorbance measured when PBS is used as a substitute for the α-glucosidase solution.

### 3.13. Anti-Inflammatory Activities

#### 3.13.1. Determination of Cell Proliferation Rate

The CCK-8 method was used to detect cell proliferation activity, with slight modifications to the previously reported method [76]. RAW264.7 cells in the logarithmic growth phase were harvested and digested with trypsin, and the cell suspension was adjusted to a concentration of 6000 cells/well. Then, 100 μL of cell suspension was added to each well of a 96-well plate and cultured in a 37 °C and 5% CO_2_ incubator for 24 h until the cells adhered. Drug treatments were applied to the cells in each group according to the experimental design. To prevent the evaporation of liquid in the outer wells of the 96-well plate, which can lead to well drying, 150 μL of PBS solution was added to the outer wells, and the drug treatment lasted 24 h. After drug treatment, the 96-well plate was removed, the original medium was discarded, and 100 μL of medium containing 10% CCK-8 reagent was added to each well. The cells were then cultured for an additional 2 h. Finally, the absorbance (OD value) of each well was measured at a wavelength of 450 nm using a microplate reader. Cell proliferation activity was calculated according to Formula (4):

(4) $$\mathrm{cell}\;\mathrm{proliferation}\;\mathrm{rate}\;(\%)=\frac{A_{s}-A_{b}}{A_{c}-A_{b}}\times 100$$

In the formula, A_s_ is the absorbance of the experimental well (containing cell culture medium, CCK-8, and the substance to be tested), A_c_ is the absorbance of the control well (containing cell culture medium, CCK-8, and no substance to be tested), and A_b_ is the absorbance of the blank well (containing cell-free culture medium and CCK-8, with no substance to be tested).

#### 3.13.2. Assay of NO Release

NO content was measured using the Griess method [77]. Groups including the normal group, model group, and polysaccharide + LPS group were set up. After centrifuging the cell culture medium at 1000 r/min for 5 min, the supernatant was collected and NO content was measured according to the instructions of the nitric oxide detection kit. In brief, RAW 264.7 cells were seeded in a 24-well plate (1 × 10^5^ cells per well). After the cells adhered, 50 μL of polysaccharide sample solution and 1 μg/mL LPS were added. After 24 h of culture, 50 μL of Griess Reagent I and 50 μL of Griess Reagent II were added. After standing for 10 min, the absorbance was measured at a wavelength of 550 nm.

#### 3.13.3. Effect of Polysaccharide Sample on the Secretion of Cytokines in RAW264.7 Cells

The levels of TNF-α, IL-1β, and IL-6 were measured using the ELISA method [78]. The specific procedure is as follows: RAW 264.7 cells were seeded in a 24-well plate, with 1 × 10^5^ cells per well, and incubated in a 37 °C and 5% CO_2_ humidified incubator for 24 h. All groups, except the normal group, were treated with LPS. The supernatant was then collected, and samples were measured according to the instructions of the ELISA kit (Enzyme Labeling Bio, Yancheng, China) to detect the levels of inflammatory cytokines (TNF-α, IL-6, and IL-1β) secreted by cells in each group. The OD values were read at 450 nm using an enzyme-labeled instrument. A standard curve was plotted using the concentration of the standard (pg/mL) on the vertical axis and the OD value on the horizontal axis, and the concentration of cytokines in each sample was calculated based on the curve equation.

### 3.14. Anti-Tumor Activity

The CCK-8 method [79] was used as previously reported to detect the proliferative inhibition effect of polysaccharide components on different cells. The test was performed on human colon cancer cells (HT-29), human liver cancer cells (HepG2), human breast cancer cells (MCF-7), and human lung cancer cells (A549). Tumor cells were seeded in a 96-well plate at a density of 1 × 10^4^ cells/mL, with a culture medium without polysaccharides as the control group. Five different concentrations of polysaccharide samples were added to the cells in the 96-well plate. The CCK-8 kit (MA0218-L-JU1-29F, Dalian Meilun Biotechnology Co., Ltd., Dalian, China) was used to measure the absorbance (OD) of each well at 450 nm to calculate cell viability.

## 4. Conclusions

This study successfully obtained polysaccharides from *Lyophyllum decastes* using ultrasound-assisted extraction (UAE) and separated them into two significantly different polysaccharide components (LDP-A1 and LDP-B1) using DEAE cellulose-52 and Sephacryl S-500. Chemical analysis and spectroscopic techniques were used to identify the primary structure of the two polysaccharides, revealing significant differences in molecular weight and monosaccharide composition. Fourier-transform infrared (FTIR) spectroscopy analysis indicated that both LDP-A1 and LDP-B1 contain β-configured glycosidic bonds. Scanning electron microscopy (SEM) observations showed distinct differences in the microstructures of the two polysaccharides, with LDP-B1 exhibiting a sheet-like structure and LDP-A1 presenting filamentous and fibrous strip forms. X-ray diffraction (XRD) analysis further revealed that LDP-B1 has an amorphous internal structure, while LDP-A1 tends towards a crystalline structure. Congo red staining confirmed that LDP-B1 possesses a typical triple-helix structure, highlighting its unique and complex structural characteristics. Biological activity tests showed that although both LDP-A1 and LDP-B1 exhibited significant antioxidant, hypoglycemic, anti-inflammatory, and anticancer activities, LDP-B1 displayed stronger biological activity in these areas, particularly with enhanced antioxidant and hypoglycemic effects. This suggests that LDP-B1 may have greater potential for developing functional foods and pharmaceuticals related to health promotion and disease prevention.

In conclusion, this study not only provides a deeper understanding of the structural characteristics and biological activity of LDPs but also offers theoretical support and practical evidence for its application in functional foods and pharmaceuticals. Given the excellent performance of LDPs in health promotion and antioxidant, hypoglycemic, and anti-inflammatory effects, we believe it has broad application prospects in related fields. Future research needs to further explore the metabolic mechanisms, pharmacological evaluation, and safety of LDPs in vivo to comprehensively assess their application potential. Currently, relevant in vivo studies are ongoing, and we hope to promote the further development and industrialization of LDPs, expanding their application in the field of natural products and providing more options for human health.

## Figures and Tables

**Table 1:** Chemical composition of purified LDP fractions.

Polysaccharide Components	Total Sugar Content	Protein Content
LDP-A1	87.21 ± 0.57%	N.D.
LDP-B1	86.47 ± 0.29%	N.D.

Note: N.D. (in the table and the following text) indicates not detected.

**Table 2:** Monosaccharide composition of LDP-A1 and LDP-B1.

Monosaccharide Composition and Content (mol %)	Different Components of Polysaccharides	
	LDP-A1	LDP-B1
Man	15.1%	8.9%
GlcA	N.D.	2.1%
Rha	0.2%	0.2%
GalA	N.D.	0.2%
Glc	40.7%	53.3%
Gal	41.5%	27.8%
Xyl	0.3%	0.5%
Ara	0.1%	0.3%
Fuc	2.1%	6.8%

## Data Availability

The data used to support the findings of this study have not been made available.
